# Holmium: YAG Popcorn Lithotripsy—In Vitro Comparison of Settings and MOSES Across Stone Densities

**DOI:** 10.1155/aiu/3584394

**Published:** 2026-08-13

**Authors:** Oleksandr Chepeliuk, Asaf Shvero, Rinat Lasmanovich, Lopez Isaias Caballero, Dina Orkin, A. Zohar Dotan, E. Dorit Zilberman, Nir Kleinmann

**Affiliations:** ^1^ Department of Urology, Sheba Medical Center, Ramat Gan, Israel, sheba.co.il; ^2^ Gray Faculty of Medical & Health Sciences, Tel Aviv University, Tel Aviv, Israel, tau.ac.il; ^3^ Operative Room Division, Anesthesia and Pain, Sheba Medical Center, Ramat Gan, Israel, sheba.co.il

**Keywords:** dusting, holmium laser, kidney stone, lithotripsy, ureteroscopy

## Abstract

**Purpose:**

To compare Holmium: YAG laser settings for popcorn lithotripsy and evaluate the impact of MOSES modulation across stone‐density groups.

**Materials and Methods:**

An in vitro bench model was used with synthetic gypsum stones prepared at three density levels (low, intermediate, and high: mean attenuation was 574, 945, and 2067 HU, respectively). For each trial, five fragments (2.8–3.3 mm) were treated using popcorn technique with Holmium: YAG laser and 272 µm fiber during a 2 min run under standardized irrigation. Five laser settings (0.3 J/80 Hz, 0.5 J/40 Hz, 1.0 J/20 Hz, 1.0 J/30 Hz, and 1.5 J/20 Hz) were tested with and without MOSES mode: each setting‐by‐modulation condition was repeated six times. The primary endpoint was percent mass reduction; secondary endpoints were residual fragment counts 1–2 and > 2 mm. Results are presented as mean ± SD, and ANOVA was used for comparison.

**Results:**

In the low‐density group, percent mass reduction was highest overall (65%–86%), with 1.0 J/30 Hz achieving the greatest reduction, MOSES provided no additional benefit (*p* = 0.6). In the intermediate‐density group, the greatest mass reduction was achieved with 1.0 J/30 Hz with MOSES (51% ± 5.9%), and MOSES effects were setting‐dependent, and at 1.0 J/30 Hz MOSES reduced total residual fragments (5.3 vs 8.0, *p* = 0.04). In the high‐density group, 1.0 J/30 Hz with MOSES achieved the highest mass reduction (71.5% ± 12.1%) and was associated with improved outcomes (*p* = 0.037). Secondary analyses of residual fragments generally paralleled these findings.

**Conclusions:**

In this in vitro popcorn lithotripsy model, 1.0 J/30 Hz provided the most consistent performance across density groups. MOSES modulation did not improve outcomes in low‐density stones but was associated with improved efficiency in intermediate‐ and high‐density stones.

## 1. Introduction

Retrograde intrarenal surgery (RIRS) and lithotripsy using high‐power Holmium: YAG (Ho: YAG) laser machines are a widespread methods for treating nephrolithiasis when appropriate [1]. The two most common methods for lithotripsy are stone “fragmentation” and stone “dusting.” The “fragmentation” method includes breaking the stone with a laser to fragments with subsequent retrieval using a basket device, and is usually performed with high‐energy, low‐frequency, and short pulse duration (0.8–2 J, 5–8 Hz, < 300 ms) [2–4]. The “Dusting” method utilizes low‐energy and high frequency (0.2–0.5 J, 15–30 Hz) and long pulse duration (> 600 ms) for lithotripsy. A complementary method for stone dusting is the “Popcorn” technique (PT). This technique includes lithotripsy in a noncontact method, with the laser fiber held in the middle of the calyx full of small‐to‐medium sized stone fragments. The PT is done using high‐energy and high‐frequency laser settings (0.5–1.5 J, 15–30 Hz) and short pulse duration (< 300 ms).

The PT was first described in 2008 as an adjunct to high‐power Ho: YAG lithotripsy [5]. This technique is used for collections of stones in a renal calyx. The laser fiber must be positioned close to the middle of the stone collection and fired continuously to induce a “whirlpool‐like” phenomenon. This causes collisions between stone fragments, which, in addition to the laser energy itself, allows for their fragmentation. Recommended laser settings for this technique were 0.5–0.8 J and 16–20 Hz [5, 6].

In the last several years, new laser machines that enable different settings with myriad adjustments such as frequency, pulse durations, and laser modulations were developed and made available and further enhanced laser energy utility for lithotripsy. The MOSES technology modulates the laser pulse into two phases: one that first creates a small vapor bubble, followed by a second pulse that transmits the remaining energy via the vapor bubble toward the target. Split‐pulse mode of MOSES technology at 20 W shows advantages for fragmenting kidney stones in popcorn lithotripsy and reduces the number of large fragments [7, 8].

Although multiple studies have evaluated the efficacy of PT, there remains no professional consensus regarding the optimal laser settings for specific clinical lithotripsy scenarios.

Accordingly, this study aimed to identify the most efficient laser parameter combinations across clinically relevant scenarios in which PT is applied.

## 2. Materials and Methods

We created an in vitro model that assessed PT within an artificial calyx, utilizing different laser settings (power, frequency, and the MOSES effect) on different stone densities. For each test, the following parameters were kept identical: laser firing time, water irrigation pressure, laser fiber caliber, laser fiber location, and number of stones. The tests were repeated over different densities of artificial stone fragments. We tested which laser settings are most efficient for different stone densities by assessing postlithotripsy fragments number, fragments size (which was checked with a filter for small particles), and remaining mass (assessed with an ultra‐low weight particles scale).

### 2.1. Stone Preparation

Artificial stones were fabricated from gypsum (Plaster of Paris) using three water‐to‐powder ratios to generate low‐, intermediate‐, and high‐density groups: 5:4 (low‐density), 5:2.5 (intermediate‐density), and 5:1 (high‐density). After 24 h of drying at 18°C, the blocks were CT‐scanned and attenuation was measured in hounsfield units (HU), yielding three blocks with distinct densities: 574 HU (low‐density), 945 HU (intermediate‐density), and 2067 HU (high‐density). The blocks were then crushed, and five fragments measuring 2.8–3.3 mm were sieve‐selected for each study set (*n* = 180). Each fragment was weighed on an analytical balance and placed into standard test tubes.

### 2.2. Popcorn Experiments

A Ho: YAG laser machine (120 W, with MOSES Version 2.0, Boston Scientific, Yokneam, Israel), was used, with a connected 272 µm laser fiber. The fiber was introduced through a dual‐lumen endourologic catheter’s main lumen, with the side lumen connected to a standard continuous irrigation system set with saline 0.9% placed at the level of 90 cm above the test tube. Stabilization of the laser was reached with a self‐made rubber plug with two side openings to make possible continuous stable irrigation. Room temperature was 18°C in all cases, and the same setup was applied for each study set. The tip of the laser fiber was placed at the distance of 3 mm from the stone mass, and continuous irrigation was initiated.

PT lithotripsy was performed continuously for a period of 2 min at laser settings of 0.3 J/80 Hz, 0.5 J/40 Hz, 1 J/20 Hz, 1 J/30 Hz, 1.5 J/20 Hz, short pulse, with and without MOSES modulation (distance mode setting, DM). 6 repetitions were performed for each intensity and MOSES effect modality, for a total of 180 study sets. For each test run, the laser fiber tip was routinely re‐cleaved to standardize a 3 mm transparent, uncoated tip length prior to firing. This step was performed as part of the protocol to ensure a consistent starting condition. Then the stone mass was dried within 6 h in a 100°C oven, followed by 3 days in room temperature of 18°C. The mass of the remaining stone was weighed, the stones were filtered with sequential sieves, and stone fragments 1–2 mm and above 2 mm were counted. Energy delivery was also documented.

We used MOSES distance mode, due to the results of a study Black et al, where MOSES effect on distance mode was found to produce the largest reduction in stone mass when using PT [8]. When not applying the MOSES effect, we used short pulse modulation, which was shown to be as effective as long pulse modulation [9].

### 2.3. Data Preparation and Statistics

The primary efficiency endpoint was percent mass reduction: secondary endpoints were residual fragment counts in the 1–2 mm and > 2 mm size categories. Analyses were performed separately for each stone‐density group to identify the most efficient power‐frequency‐modulation combination. Continuous outcomes are reported as mean ± SD, unless otherwise stated. Differences across laser conditions (five settings tested with and without MOSES) were assessed using one‐way analysis of variance (ANOVA) within each density group. A two‐sided *p* < 0.05 was considered statistically significant. Statistical analyses were performed using SPSS (Version 26.0; IBM Corp., Armonk, NY, USA).

### 2.4. Ethical Considerations

Institutional review board approval was not required, as all experiments were performed under controlled in vitro laboratory conditions without human or animal subjects or personal data involvement.

## 3. Results

Overall, 180 study repetitions were performed, 60 rounds in each stone density group: low‐, intermediate‐ and high‐density stones, 6 times for each laser setting, including the use of MOSES effect.

### 3.1. Low‐Density Stone Group

**FIGURE 1:**
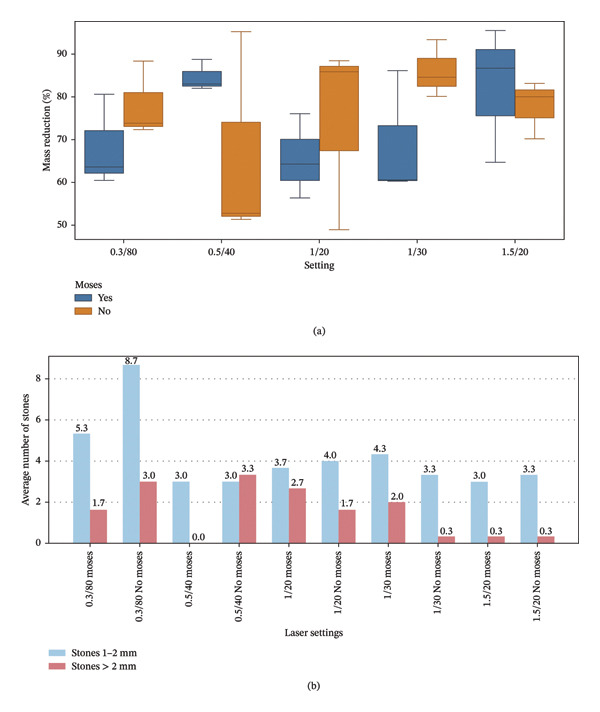
Low‐density stones. (a) Mass reduction by laser setting and MOSES effect. Percent mass reduction by laser setting and MOSES mode. Boxplots display the median (IQR) with whiskers indicating the data range: *n* = 3 runs per condition. Mass reduction is expressed in percentages (*Y*‐axis). *X*‐axis demonstrates joule and Hz units. For consistency with the text, summary values in the results are reported as mean ± SD. (b) Residual fragments count by laser setting and MOSES mode. Bars show the average number of residual fragments in the 1–2 mm and > 2 mm size categories after popcorn lithotripsy for each setting. Values above bars denote means.

Using percent mass reduction as the primary efficiency endpoint, the average mass reduction ranged from 65%–86% across settings. There was no statistically significant difference between MOSES and non‐MOSES modes (*p* = 0.6). The highest average mass reduction was achieved with 1 J/30 Hz without MOSES (86.1% ± 6.7%), followed by 0.5 J/40 Hz with MOSES (84.5% ± 3.6%), and 1.5 J/20 Hz with MOSES (82.2% ± 15.8%) (Figure 1a). For the secondary endpoint, 0.3 J/80 Hz without MOSES produced the highest residual burden (8.7 ± 2.3 fragments 1–2 mm and 3.0 ± 5.2 fragments > 2 mm) (Figure 1b). The lowest average number of 1–2 mm fragments (3.0) was observed with 0.5 J/40 Hz (with and without MOSES) and 1.5 J/20 Hz with MOSES, whereas the lowest average number of > 2 mm fragments was observed with 0.5 J/40 Hz with MOSES (0.0). Overall differences between laser settings were statistically significant (*p* < 0.05).

### 3.2. Intermediate‐Density Stone Group

**FIGURE 2:**
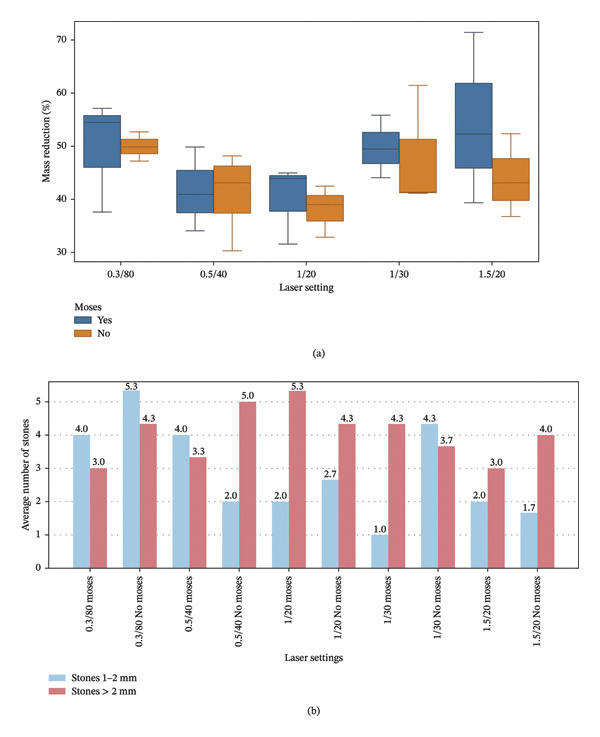
Intermediate‐density stones. (a) Mass reduction by laser setting and MOSES effect. Percent mass reduction by laser setting and MOSES mode. Mass reduction is expressed in percentages (*Y*‐axis). *X*‐axis demonstrates joule and Hz units. For consistency with the text, summary values in the results are reported as mean ± SD. (b) Residual fragments count by laser setting and MOSES mode. Bars show the average number of residual fragments in the 1–2 mm and > 2 mm size categories after popcorn lithotripsy for each setting. Values above bars denote means.

For the primary efficiency endpoint, 1.0 J/30 Hz with MOSES achieved the highest average mass reduction (51% ± 5.9%), followed by 0.3 J/80 Hz without MOSES (50.0% ± 2.8%) (Figure 2a). Overall, intermediate‐density stones showed lower efficiency than low‐density stones, reflected by both reduced mass reduction and a higher number of residual fragments. For the secondary endpoint, 1.5 J/20 Hz with MOSES yielded relatively low residual counts (2.0 ± 1.0 fragments 1–2 mm and 3.0 ± 2.6 fragments > 2 mm), whereas 0.3 J/80 Hz without MOSES produced the highest residual burden (5.3 ± 3.2 fragments 1–2 mm and 4.3 ± 0.6 fragments > 2 mm) (Figure 2b). Differences in residual fragment burden across laser settings were significant (*p* < 0.05). MOSES effects were setting dependent and appeared to shift the fragment‐size distribution: at 1.0 J/30 Hz, MOSES reduced the total residual fragment burden (5.3 vs 8.0 fragments, *p* = 0.04), largely due to fewer 1–2 mm fragments (1.0 vs 4.3), whereas > 2 mm fragment counts were comparable (4.3 vs 3.7).

### 3.3. High‐Density Stone Group

**FIGURE 3:**
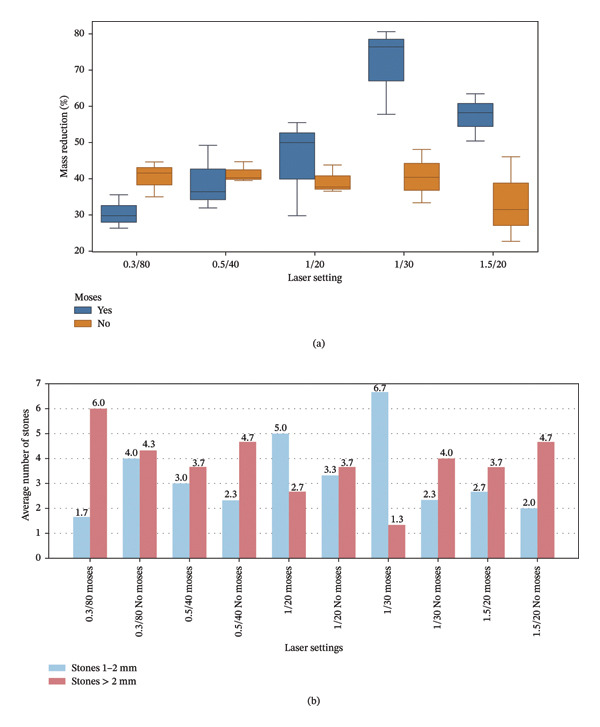
High‐density stones. (a) Mass reduction by laser setting and MOSES effect. Percent mass reduction by laser setting and MOSES mode. Mass reduction is expressed in percentages (*Y*‐axis). *X*‐axis demonstrates joule and Hz units. For consistency with the text, summary values in the results are reported as mean ± SD. (b) Residual fragments count by laser setting and MOSES mode. Bars show the average number of residual fragments in the 1–2 mm and > 2 mm size categories after popcorn lithotripsy for each setting. Values above bars denote means.

For the primary efficiency endpoint, 1.0 J/30 Hz with MOSES achieved the highest average mass reduction (71.5% ± 12.1%). The use of MOSES was associated with a significant improvement in outcomes in this group (*p* = 0.037) (Figure 3a). For the secondary endpoint, the lowest total fragment burden was observed with 1.5 J/20 Hz with MOSES (2.7 ± 1.2 fragments 1–2 mm and 3.7 ± 1.5 fragments > 2 mm: total 6.3) and 1.0 J/30 Hz without MOSES (2.3 ± 3.2 fragments 1–2 mm and 4.0 ± 1.7 fragments > 2 mm: total 6.3) (Figure 3b). The highest residual burden occurred with 0.3 J/80 Hz without MOSES (4.0 ± 3.6 fragments 1–2 mm and 4.3 ± 2.1 fragments > 2 mm: total 8.3).

## 4. Discussion

Nowadays several laser lithotripsy strategies are used, including dusting, fragmentation, painting, chipping, and dancing [10]. After initial fragmentation into smaller pieces, the PT can further reduce fragment size and number by using high‐frequency and high‐power settings to generate a noncontact, whirlpool‐like effect [5, 11].

In this study, we measured quantitative in vitro lithotripsy outcomes across stones of different densities, comparing commonly used laser settings and assessing the contribution of MOSES distance mode.

As expected, stone mass reduction was greatest in the low‐density group (65%–86%), and MOSES did not provide additional benefit (*p* = 0.6). The best setting for low‐density stones was 1 J/30 Hz without the MOSES effect, with an average of 3.3 stone fragments 1–2 mm left after applying PT. Although these stones are generally easier to treat with Ho: YAG, higher energies still improved reduction.

In the intermediate‐density group, mass reduction was lower (51% ± 5.9%), and the most efficient setting was again 1 J/30 Hz. MOSES modulation was associated with a reduction in total residual fragments count (*p* = 0.04).

In the high‐density stone group, 1 J/30 Hz with MOSES achieved the greatest mass reduction and improved fragmentation (71.5% ± 12.1%, *p* = 0.037), leaving a low average of clinically relevant fragments of 1.3 ± 1.5 > 2 mm. Overall, using mass reduction as the efficiency endpoint, 1 J/30 Hz was the most effective popcorn setting across stone densities and can therefore be applied broadly. However, high‐power holmium use in the calyceal system (e.g., > 40 W) may raise concerns for thermal injury: reassuringly, thermal thresholds were not reached when high irrigation rates were used [12]. Use of a ureteral access sheath further increases outflow, improves operative‐field cooling, and supports safer use of higher‐power settings [13].

Notably, mass reduction at 1.0 J/30 Hz with MOSES was higher in the high‐density than in the intermediate‐density group. This likely reflects that HU is an imperfect surrogate for comminution: fragmentation depends on internal structure and mechanical properties (e.g., porosity/fracture behavior) that may not track linearly with attenuation [14, 15]. Artificial stone formulations can differ in mechanical behavior beyond their measured attenuation, and changes in mixture/water content are known to alter tensile strength, potentially affecting comminution efficiency [16]. In addition, popcorn efficiency depends on fragment dynamics and interaction conditions (e.g., contact/noncontact behavior and local geometry), which can amplify formulation‐dependent differences in how fragments circulate and re‐enter the laser field [9].

### 4.1. MOSES Effect

In intermediate‐ and high‐density stones, MOSES improved stone mass reduction and reduced the size and number of residual fragments, which may facilitate fragments washout while using ureteral access sheath or spontaneous passage after RIRS. This may be a result of the better energy delivery produced by the MOSES effect, which may enhance both the direct energy effect on the stones and contribute to the whirlpool effect. In addition, the MOSES effect was shown to have a protective effect of the second vaporization bubble which collapses away from the fiber tip, reduces fiber degradation, and therefore enhances stone ablation efficiency [8].

### 4.2. Fiber Degradation

With respect to pulse length, long pulse has been shown to result in less fiber tip degradation than short pulse at 20, 35, and 40 W [18]. We tested only a short pulse to maximize power delivery to fragments. No additional (unplanned) re‐cleaving was required due to observable degradation during the test runs, suggesting that clinically meaningful tip wear was limited under our experimental conditions. The use of a 272 µm fiber and the shorter firing duration (2 vs 4 min) may also have mitigated degradation compared with prior work [17, 18]. Using identical test tubes and a fixed irrigation rate helped emulate the renal calyx and the RIRS environment [19].

Comparison with prior work is summarized in Table 1. Chawla et al. were the first to validate the PT in an in vitro caliceal model and reported maximal stone reduction over a 2 min run using 1.5 J/40 Hz with a 365 µm fiber [5]. However, this high‐power, high‐frequency combination may be associated with increased heat generation and greater risk of fiber burnback, particularly if irrigation is suboptimal. For this reason, and to focus on settings that are more practical for flexible ureteroscopy, we did not test 1.5 J/40 Hz. In addition, the use of a large‐caliber 365 µm fiber can reduce scope deflection and constrain irrigation flow, which may further limit the feasibility and safety margin of high‐energy popcorn in vivo.

**TABLE 1:** Comparison of popcorn laser settings.

Author (year)	Distance	Fiber (μm)	Stone types (*N*) and density	Laser fire time	Total tests (*N*)	Laser settings	Best suggestions, comments
Chawla (2008) [5]	2 mm	365	Soda‐lime (4)	2 min	60	1 J and 20, 30, 40 Hz	1.5 J/40 Hz, old generation laser machine
Kronenberg (2017) [11]	1 mm	275, 365	Bego stones (4), 1900 HU	2 and 4 min	144	0.5 J and 10, 20, 40 Hz (SP or LP); 1.5 J and 10, 20, 40 Hz (SP or LP)	1.5 J/20 Hz, 273 μm laser fiber, > 4 min laser fire time
Aldoukhi (2018) [9]	0–2 mm	242	Bego stones,	3 and 6 min	140	1 J/20 Hz, 0.5 J/40 Hz, 1 J/40 Hz (SP or LP); 0.5 J/80 Hz (SP only)	0.5 J/80 Hz (SP) for 3 min, 0 mm fiber stone distance, no > 2 mm fragments
Jongjitaree and Chotikawanich (2019) [20]	Not specified (caliceal model)	200	Panamix, gypsum, Plaster of Paris	> 5 min (5.13–17.56 min)	30	1 J/20 Hz, 2 J/10 Hz, 1.5 J/20 Hz, 3 J/10 Hz, 2 J/20 Hz	Optimal setting: 2 J/20 Hz (yielded highest vaporization % and fastest times: 8:51 min for hard stones, 5:13 min for soft stones)
Current study	3 mm	272	Plaster of Paris (5), 574 HU, 945 HU, 2067 HU	2 min	180	0.3 J/80 Hz, 0.5 J/40 Hz, 1 J/20 Hz, 1 J/30 Hz, 1.5 J/20 Hz‐(SP), with or without MOSES (DM) for 3 density types.	1 J/30 Hz (SP) for all stone types, add MOSES mode (DM) for hard stones (2067 HU) add value to efficacy of popcorning (*p* = 0.037)

Abbreviations: DM, distance mode; HU, hounsfield units; LP, long pulse; SP, short pulse.

Other published studies have proposed a range of popcorn settings, most commonly within 0.5–1.0 J and 16–30 Hz [10, 21]. Emiliani et al. reported efficient noncontact fragmentation using 1.5 J/20 Hz with a 273 µm fiber, with the goal of generating clinically insignificant fragments. However, their experiments were performed using a single high‐density stone model (1900 HU), which makes it difficult to extrapolate performance across the broad spectrum of stone densities encountered in clinical practice [22, 23]. Spore et al. demonstrated shorter Ho: YAG lithotripsy times with higher power settings, but their work largely evaluated contact lithotripsy of a single stone and did not focus on multifragment, noncontact “distant” popcorn environments [24]. The key advance of the present study is the use of an HU‐stratified in vitro model spanning low‐, intermediate‐, and high‐density stones and the inclusion of pulse modulation (MOSES distance mode) as an explicit experimental factor, enabling density‐specific assessment of both setting selection and modulation effects. This approach allows a practical conclusion: 1.0 J/30 Hz performs consistently across density groups, while MOSES modulation provides incremental benefit predominantly in higher‐density stones, reflected in both residual mass and clinically relevant fragment‐size distributions.

In recent years, there has been growing interest in advanced suction‐assisted technologies, including flexible and navigable suction ureteral access sheaths (FANS). These novel systems introduce active or semi‐active aspiration combined with increased sheath flexibility, allowing improved conformability within the collecting system. This evolution has led to the emergence of FANS‐assisted flexible ureteroscopy (FANS f‐URS), which has been associated with enhanced stone‐free rates, improved fragment evacuation, and reduced intrarenal pressure compared to conventional UAS systems. Many contemporary endourologists now preferentially dust stones to a small residual burden and subsequently remove fragments using suction‐assisted techniques, with popcorn lithotripsy serving primarily as an adjunct when fragment clearance is incomplete or suction devices cannot be effectively deployed [25].

While this work offers density‐stratified guidance for popcorn settings and evaluates the incremental effect of MOSES modulation, several limitations should be considered. Firstly, this was an in vitro study and therefore cannot fully replicate the complex in vivo environment of lithotripsy (e.g., variable irrigation, temperature dynamics, and stone mobility). Second, our findings are based on a single holmium laser platform and the specific pulse and frequency settings available on that system, which may limit generalizability to other generators. Third, to standardize comparisons across densities, we used manufactured stone phantoms; although designed to approximate different HU ranges, these models cannot fully reproduce the heterogeneous composition and fragmentation behavior of human urinary calculi. Nevertheless, our results provide practical, density‐stratified guidance for selecting holmium laser settings when applying the PT.

Further research is needed to evaluate key parameters for clinical translation, including intrarenal pressure, temperature elevation, fiber degradation, and potential effects on renal tissue. In addition, outcomes should be assessed across different stone compositions (e.g., calcium oxalate monohydrate, calcium oxalate dihydrate, uric acid, and infection stones) to better define setting selection and inform clinical decision‐making.

## 5. Conclusions

Popcorn lithotripsy is a practical adjunct to flexible ureteroscopy for reducing residual fragments after initial fragmentation. In this in vitro, HU‐stratified model, 1.0 J/30 Hz showed the most consistent performance across stone densities, supporting it as a broadly applicable default popcorn setting during RIRS. MOSES DM provided no added benefit in low‐density stones but improved efficiency in denser stones in a setting‐dependent manner, including improved mass reduction in high‐density stones. Clinically, a simple approach is to use 1.0 J/30 Hz for popcorn and consider MOSES when treating intermediate‐ to high‐density stones to enhance efficiency without escalating energy.

## Funding

No funding was received for this manuscript.

## Conflicts of Interest

The authors declare no conflicts of interest.

## Data Availability

The data that support the findings of this study are available from the corresponding author upon reasonable request.
